# Geographical Origin Discrimination of Monofloral Honeys by Direct Analysis in Real Time Ionization-High Resolution Mass Spectrometry (DART-HRMS)

**DOI:** 10.3390/foods9091205

**Published:** 2020-09-01

**Authors:** Vincenzo Lippolis, Elisabetta De Angelis, Giuseppina Maria Fiorino, Annalisa Di Gioia, Marco Arlorio, Antonio Francesco Logrieco, Linda Monaci

**Affiliations:** 1 Institute of Sciences of Food Production (ISPA), National Research Council of Italy (CNR), Via G. Amendola 122/O, 70126 Bari, Italy; elisabetta.deangelis@ispa.cnr.it (E.D.A.); giuseppina.fiorino@outlook.com (G.M.F.); annalisadigioia@hotmail.it (A.D.G.); antonio.logrieco@ispa.cnr.it (A.F.L.); linda.monaci@ispa.cnr.it (L.M.); 2 Dipartimento di Scienze del Farmaco, Università degli Studi del Piemonte Orientale “Amedeo Avogadro” (UNIUPO), Largo Donegani 2, 28100 Novara, Italy; marco.arlorio@uniupo.it

**Keywords:** monofloral honey, direct analysis in real time (DART), high resolution mass spectrometry (HRMS), geographical origin, chemometrics

## Abstract

An untargeted method using direct analysis in real time and high resolution mass spectrometry (DART-HRMS) combined to multivariate statistical analysis was developed for the discrimination of two monofloral (chestnut and acacia) honeys for their geographical origins—i.e., Italy and Portugal for chestnut honey and Italy and China for acacia honey. Principal Component Analysis, used as an unsupervised approach, showed samples of clusterization for chestnut honey samples, while overlapping regions were observed for acacia honeys. Three supervised statistical approaches, such as Principal Components—Linear Discriminant Analysis, Partial Least Squares—Discriminant Analysis and k-nearest neighbors, were tested on the dataset gathered and relevant performances were compared. All tested statistical approaches provided comparable prediction abilities in cross-validation and external validation with mean values falling between 89.2–98.4% for chestnut and between 85.8–95.0% for acacia honey. The results obtained herein indicate the feasibility of the DART-HRMS approach in combination with chemometrics for the rapid authentication of honey’s geographical origin.

## 1. Introduction

Honey is a complex and high-quality natural product containing a wide range of nutritional and therapeutic properties but with a limited production and high commercial prices. Honey is defined by European Union legislation as the natural sweet substance produced by bees of *Apis mellifera* species from nectar or sugary secretions of plants, as well as from excretions of plant-sucking insects on the living parts of plants [1]. Both the European Union and Codex Alimentarius laws establish that the geographical origin, in terms of country of production, must be indicated on the label, also supplemented by specific reference to the floral or vegetable origin. Moreover, in the case of blends of honey, their origin should be declared as a “blend of EC honeys”, “blend of non-EC honeys” or “blend of EC and non-EC honeys” [1,2].

Geographical and botanical origins of honey account for the peculiar chemical composition and organoleptic characteristics of the final product [3,4]. Monofloral honeys, mostly deriving from a single plant species (at least 45% of pollen grains), may considerably differ in their sensory properties with highly prominent flavor and aroma. Acacia (*Robinia pseudoacacia*) honey is one of the most consumed monofloral honeys in Europe, being appreciated for its permanently liquid state, light color, floral aroma and sweet and delicate taste [5]. Similarly, chestnut (*Castanea sativa*) honey is considered one of the most delicious and high-quality honeys, being a very good source for nectar and pollen [6,7]. For these reasons, monofloral honeys, and in particular those derived from acacia and chestnut, have recently gained consumer preferences, with an increased demand and commercial value [4]. Due to their increased commercial value, monofloral honeys are highly susceptible to fraudulent practices through mislabeling and mixing with cheaper and lower-quality honeys or with various sugar syrups.

Honey is produced in different areas of the world, with more than 2.3 million tonnes produced worldwide in 2018, with China and Turkey as main producers [8]. China is also the largest exporter of honeys in the world, while in Europe, Portugal is the country bearing the highest number of geographical protected labels on honey [9]. Honey composition is quite variable and strictly linked to its floral source and geographical origin, but external factors, including processing, packaging and storage conditions, could play an important role. Although Italy is one of the EU countries with the highest honey production [10], the market demand for honey is higher than domestic production, therefore a substantial amount of honey is imported from elsewhere in Europe and from third-world countries, in which production does not always meet the high food safety standards required. This can lead to honey mislabeled with regard to its geographical and/or botanical origin [11].

The traceability certifying the geographical origin of food products is of primary importance for traders and producers, as well as to reinforce consumer trust. The complex task of the determination of food origin is commonly applied to control products in both customs control and self-control programs of the food industry.

Melissopalynological analysis of pollen is the most used approach for the botanical and geographical origin classification of honey, as the pollen spectrum is strictly related to the environment where the nectar is collected [12]. This analysis is often complemented by other analytical methodologies, mainly based on chromatographic techniques, to assure the honey authenticity [13]. Often, the use of conventional and targeted methods is time-consuming and not sufficient to guarantee the evaluation of complex matrices, including honeys. For this reason, the development of rapid and reliable non-targeted analytical approaches, such as fingerprinting and profiling methods, is highly demanded. Indeed, these methods combined to chemometric tools allow for the detection of a high number of metabolites, leading to samples based on their pattern.

Several analytical techniques, mainly based on nuclear magnetic resonance [5,14,15], Raman and infrared spectroscopy [16,17], mass spectrometry [18,19,20], electronic tongue [21,22] and electronic nose [23,24], in combination with chemometrics, have been applied to discriminate the geographical origin of honey.

The use of ambient mass spectrometry (AMS) is continuously increasing in the field of metabolomic fingerprinting as a high-throughput alternative to more traditional hyphenated methods for authentication issues [25]. Among AMS techniques, direct analysis in real-time mass spectrometry (DART-MS), being simple and requiring a very limited sample preparation, has been shown to be the most promising and versatile technique, proving to be a rapid tool in the assessment of food authenticity and food quality, also thanks to the use of fast and streamlined protocols [25,26,27]. Such an approach offers several advantages over the conventional techniques, including direct sample analysis in open atmosphere, high sample throughput and minimal or no sample preparation requirements, the soft ionization of a wide range of both polar and apolar compounds. Several papers have been recently published demonstrating the applicability of DART-MS to assess food authenticity and detect food adulterations of olive oil [28], beer [29], wine [30], animal fat [27], milk [31] and salmon [32]. Only one paper reported the applicability of DART-MS to the discrimination of geographical origin of food—i.e., garlic produced in Czech Republic, Spain and China [33]. Regarding honey products, DART-HRMS was used as alternative approach for the determination of 5-hydroxymethylfurfural [34,35]. To the best of our knowledge, no studies based on DART-MS have been performed to date for the assessment of geographical origin of honeys.

In this context, the aim of this study was to demonstrate the feasibility of the DART-HRMS technique for the discrimination of the geographical origin of honeys. Specifically, a rapid and suitable non-targeted DART-HRMS method in combination with multivariate statistical analysis was developed and validated to discriminate two monofloral honeys varieties (i.e., chestnut and acacia) for their geographical origin (i.e., Italy and Portugal and Italy and China, respectively). Different statistical classification models were investigated and applied to the analysis of honey samples and performance results were compared.

## 2. Materials and Methods

### 2.1. Chemicals and Reagents

Methanol (HPLC grade) was purchased from Sigma-Aldrich (Milan, Italy). Ultrapure water was produced by a Milli-Q^®^ Direct system (Merck KGaA, Darmstadt, Germany). Helium (99.9995% purity) was provided by Sapio S.r.l. (Bari, Italy). Regenerate cellulose (RC) syringe filters with 0.2 µm of porosity were purchased by from VWR International (Milan, Italy). OpenSpot (OS) Sample Cards were purchased by Ion Sense Inc. (Saugus, MA, USA).

### 2.2. Honey Samples

A total of 234 monofloral honey samples commonly found in marketplaces and collected in different countries with certified origins were selected for this study. Specifically, 117 chestnut honey samples were collected from Italy (39) and Portugal (78), while 117 acacia honey samples were collected from Italy (78) and China (39). The authenticity of the monofloral honeys was assessed by internal certified protocols performed by Coop Italia Soc. Cooperativa (Casalecchio di Reno, Italy) which provided samples. Only honey samples produced in seasons 2017–2018 were taken into account.

### 2.3. Sample Preparation

Sampling and homogenization of honey samples were performed according to AOAC 920.180 protocol [36]. For sample preparation, a rapid protocol aimed at retaining as many honey metabolites as possible—thus to obtain most comprehensive spectra applicable for discriminating between different geographical origin—was optimized for the DART-HRMS analysis. In particular, an aliquot (1 g) of homogenized honey was added to a mixture of MeOH/H_2_O (1:1, *v/v*), (50 mL) and the sample was vortexed for 3 min. After filtration using 0.2 µm RC syringe filter, the filtered extract was directly analyzed by DART-HRMS.

### 2.4. DART-HRMS Analysis

DART-HRMS analyses were carried out by using a DART ionization source SI-140-GIST (DART Thermo Ion Max Vapur Interface, Ion Sense Inc., Saugus, MA, USA) coupled to an Exactive™ monostage Orbitrap™ High Resolution mass spectrometer (Thermo Fisher Scientific, San Jose, CA, USA). An aliquot (2 μL) of the honey extract was placed onto the metallic grid of the OpenSpot^®^ sample cards and kept at 60 °C for 5 min to facilitate solvent evaporation before its introduction into the DART source holder. The operating conditions of the DART source were: positive ion mode; helium flow of 3.2 L/min for 1 min and heated at 250 °C; discharge needle voltage kept at −6 kV; grid electrode voltage set to 250 V; distance between DART exit and MS inlet set at 5 mm. The operating conditions of DART source were set by DART-SVP controller (v. 4.0.x). The main settings of the Exactive™ mass spectrometer were the following: mass scan range of 100–600 m/z; resolution set at 25,000 (FWHM at m/z 200); microscan number of 4; Automatic Gain Control (AGC) Target of 3 × 10^−6^; maximum injection time (IT) of 250 ms; capillary voltage set to 30 V; tube lens voltage set to 65 V; capillary temperature kept at 250 °C. Calibrations of the MS system were periodically performed by the direct infusion ESI-MS approach of the positive ion calibrating solution, provided by the manufacturer, in order to obtain a mass accuracy lower than 5 ppm. The MS system was controlled by using the Xcalibur™ v. 2.1 software (Thermo Fisher Scientific, San Jose, CA, USA).

To carry out the subtraction of the spectral background, a blank open spot card was acquired before analyzing each sample by DART-HRMS acquiring the relevant spectrum for 30 s.

### 2.5. Data Processing and Statistical Analysis

In the first step of data processing, DART-HRMS spectra acquired in the time range of 30 s were averaged and then subtracted of spectral background by using the Xcalibur™ software. Successively, for each honey sample, the full list of accurate m/z ratios and peak intensities obtained was exported and processed by MetaboAnalyst 3.0 (http://www.metaboanalyst.ca/) [37,38] for peak matching and alignment with mass tolerance of 0.25, imputation of missing values (replacing missing elements by using the half of the lowest measured peak intensity) and data filtering (by using Interquartile Range approach). Successively, after pre-processing obtained by data centering, the dataset was submitted to multivariate statistical analyses performed by V-Parvus software (release 2010, http://www.parvus.unige.it, Genova, Italy).

Principal Component Analysis (PCA) was used as an unsupervised technique to evaluate the presence of outliers. Specifically, PCA was applied to each single group of monofloral honey samples of different geographical origin, observing the relevant influence plots and excluding samples identified as extreme outliers. To establish the exact number of Principal Components (PCs) to be used to build PCA models, the Non-linear Iterative Partial Least Squares (NIPALS) algorithm was applied using V-fold of 10 (cross validation process, CV = 10). PCA was also used as exploratory technique to visualize the presence of natural sample clustering between monofloral honey samples in relation to their geographical origin [39].

Afterwards, three supervised pattern recognition techniques—i.e., Linear Discriminant Analysis (LDA), Partial Least Squares Discriminant Analysis (PLS-DA) and k-nearest neighbors (k-NN) [40], were exploited to classify monofloral honey samples on the basis of their geographical origin. For this purpose, the two data matrices were randomly split in two subsets: a modelling set (containing 60 samples) and a test set (containing 57 samples). Specifically, for each monofloral honey, a modelling set, composed by 30 samples for each geographical origin, was used to build the three different statistical models. Test sets, consisting of 9 Italian and 48 Portuguese chestnut honey samples and 48 Italian and 9 Chinese acacia honey samples, were used for the validation process. 

The chemometric models of PCA-LDA was built by firstly performing PCA test to reduce the number of variables that exceeded the number of objects, thus preventing model overfitting; then the selected scores were used as classification variables for LDA [41,42]. Indeed, the number of variables should not exceed (n-g)/3, where n is the number of objects and g is the number of categories. Considering that modelling sets were composed by 60 objects (number of samples) and 2 categories (number of geographical origins) the maximum number of variables should be approximately 19.

The appropriate numbers of principal components, latent variables and k values, respectively, for PCA-LDA, PLS-DA and k-NN models were established by evaluating those determining the lowest prediction error rate in cross-validation (cross-validation segments, V = 10). This parameter guarantees to improve feature variables and, at the same time, to avoid model overfitting. Model performances for PCA-LDA, PLS-DA and k-NN, expressed as percentages, were compared with reference to their recognition ability—i.e., the ability to correctly classify samples of the modelling set—prediction ability in cross-validation (CV)—i.e., the ability to correctly classify samples of a test set generated in a V-fold cross validation—and prediction ability in external validation—i.e., the ability to correctly classify samples of the test set.

## 3. Results and Discussion

In the present study, the real-time mass spectrometry (DART-MS) combined with chemometric analysis, was used for the first time to the discrimination of two kind of monofloral honey samples, namely chestnut and acacia, based on their geographical origin. As for chestnut, Italian and Portuguese honey samples were compared to each other, while for acacia, Italian honeys were compared with samples from China.

**Figure 1:**
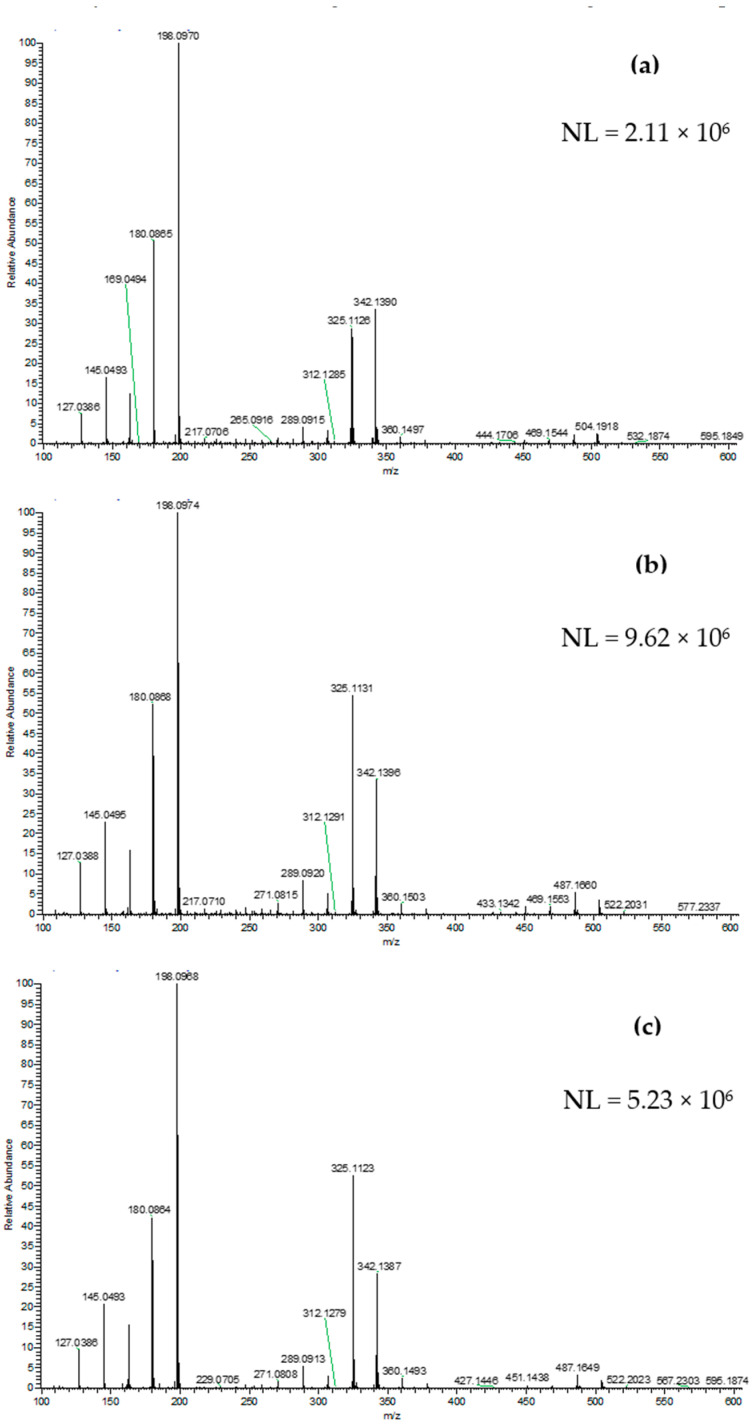

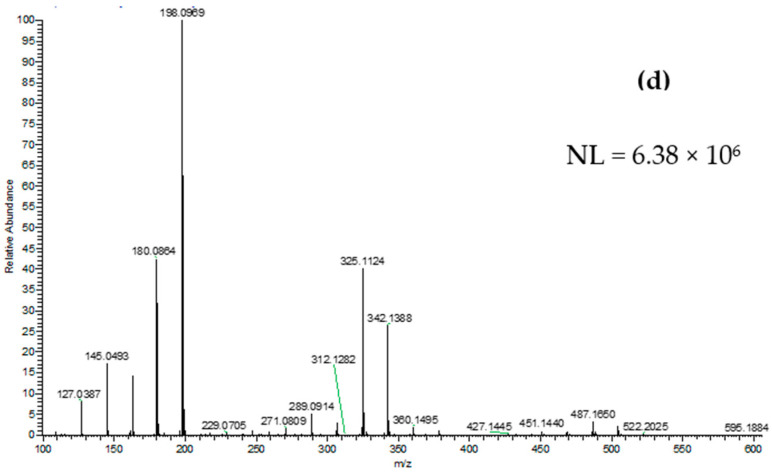
Representative DART-HRMS positive ion spectra acquired for the sample extracts of chestnut honeys from Italy (**a**), chestnut honeys from Portugal (**b**), acacia honeys from Italy (**c**) and acacia honeys from China (**d**). NL: Normalization level.

Figure 1 reports four representative DART-HRMS average spectra, after blank subtraction, obtained for the chestnut honey extracts of Italian (Figure 1a) and Portuguese (Figure 1b) samples and acacia honey extracts of Italian (Figure 1c) and Chinese (Figure 1c) samples. 

At first, a preliminary PCA was performed on pre-processed spectra of chestnut and acacia honey samples in order to explore the presence of outlier samples. PCA score plots highlighted that, in the case of chestnut samples, seven PCs described 96.3% of total variance for samples from Italy while nine PCs described 93.0% of total variance for samples from Portugal. In the case of acacia honey samples, PCA models showed that eight PCs described 93.7% of total variance for samples from Italy while nine PCs described 91.0% of total variance for samples from China. The absence of outliers in all classes was demonstrated using influence plots where the Mahalanobis distance was plotted versus sample residual.

**Figure 2:**
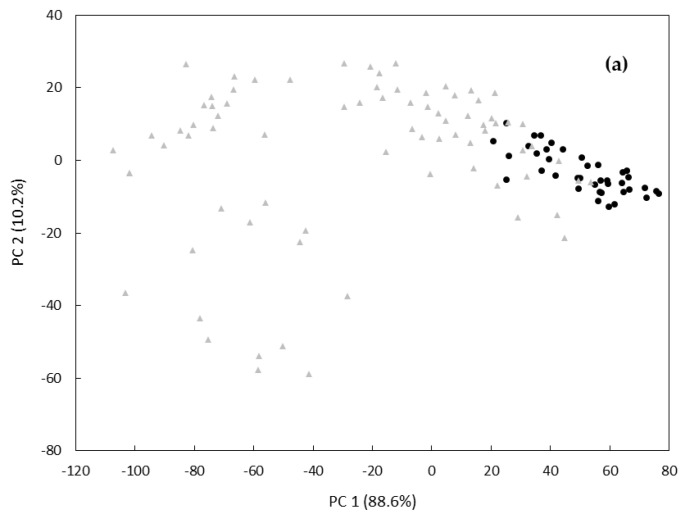

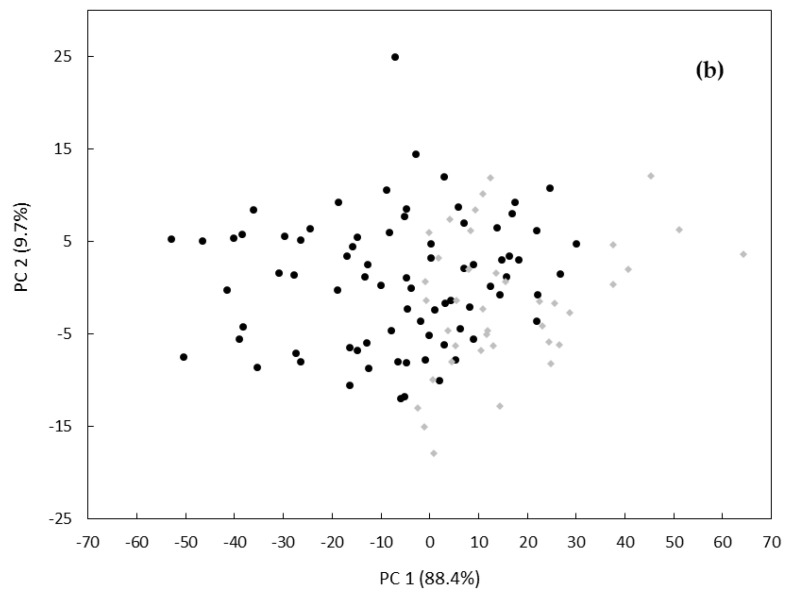
PC1 vs. PC2 scatter plots for monofloral chestnut (**a**) and acacia (**b**) honey samples. Geographical origins: Italy (black filled circle), Portugal (grey filled triangle), China (grey filled rhombus).

Subsequently, an explorative PCA was performed using the entire data set to obtain an overview of the data distribution for each monofloral honey. Figure 2 shows the PCA score plot (PC1 vs. PC2) obtained for chestnut honey samples (Figure 2a) and for acacia honey samples (Figure 2b). A discrete visual clustering of the objects on the basis of their geographical origin was observed for chestnut honeys (PC1 and PC2 explained 88.6% and 10.2% of the total variance, respectively), while overlapping regions were observed for acacia honeys with a modest clustering for their geographical origin (with 88.4% and 9.7% of the total variance explained by PC1 and PC2, respectively). Additionally, by analyzing the score plots of the remaining PCs no visual clusterization was observed.

These results were confirmed by analyzing the Fisher weight (FW) values of the principal components, which measure the between-class variance/within-class variance ratio. Indeed, FW values resulted to be 2.64 for the PC1 of chestnut honeys samples and lower than 1 for all the remaining PCs of both data sets (data not shown). These results indicated that the PCA was not able to discriminate honey samples on the basis of their geographical origin; therefore, it was necessary to treat data with three different supervised discriminant techniques—i.e., PCA-LDA, PLS-DA and k-NN. These classification techniques were tested on both chestnut and acacia honey samples previously split into two subsets: a modeling set and a test set. Overall, results are indicated in Table 1 and Table 2, for chestnut and acacia honeys, respectively.

As for LDA, PCA was used as strategy for variable reduction and to avoid model overfitting. The number of PCs (seven and nine for chestnut and acacia honeys, respectively) to be used to build the PCA-LDA models was selected on the basis of the error in prediction cross validation that has to be the lowest (CV procedure with V = 10). The PCA-LDA models provided mean value of recognition ability of 98.4% for chestnut honeys (Table 1) in both classification and CV prediction and 95.0 and 93.4% for acacia honeys (Table 2) in classification and CV prediction, respectively. The model applicability was also tested by using the test set providing mean prediction abilities of 90.3 and 89.2%, for chestnut and acacia honeys, respectively (Table 1 and Table 2).

PLS-DA was applied as an alternative multivariate statistical approach of classification offering the advantage to avoid variables reduction processes. Specifically, by applying a 10-fold cross-validation, 10 and 12 latent variables (LVs) were found to produce the optimal model complexity for chestnut and acacia honey data sets, respectively. In these conditions, mean recognition rates were higher than 96.7% in both cases (Table 1 and Table 2). Specifically, all Italian samples was correctly classified, while one Portuguese and two Chinese samples were not correctly assigned. The mean CV prediction rates were 96.7 and 95.0%, for chestnut and acacia honeys, respectively. In addition, mean prediction abilities of 89.2% and 85.8% for chestnut and acacia honeys samples, respectively, were obtained for the external validation procedure (Table 1 and Table 2). 

In the case of k-NN, the prediction error rate in cross-validation (V = 10) was calculated for each different k value. The smallest k value determining the lowest error was 3 for both data sets and therefore it was selected as the optimal value. The k-NN models provided mean recognition abilities in the range between 95.0–98.4%, while CV predictions were of 98.4 and 91.7%, for chestnut and acacia honeys samples, respectively. Finally, mean prediction abilities of 91.4% and 90.3% were obtained in the external validation for chestnut and acacia honeys, respectively.

The results herein obtained were in accordance with a similar study focused on the geographical authentication of Italian honey based on an NMR-metabolomic approach [5]. The authors developed a PLS2-DA model able to correctly discriminate 100% of Italian honeys from Eastern European ones. In another study, MIR analysis in combination with a PCA-LDA model were found able to distinguish geographical origins of monofloral honeys from Switzerland, Germany, and France, with prediction abilities ranged from 76 to 100% [17] although only a limited number of samples was used for the analysis.

In the current study, the DART-HRMS untargeted approach coupled with three supervised techniques, such as PCA-LDA, PLS-DA and k-NN, were investigated for discriminating Italian chestnut and acacia honey from Portuguese and Chinese samples. The results showed that all developed models provided acceptable and comparable prediction abilities, highlighting the robustness of the entire method, its applicability being unaffected by the statistical approach used to assess the authenticity of unknown samples. Moreover, these results demonstrated that DART-HRMS technique provides informative experimental data useful to build up appropriate models for the discrimination of monofloral honey samples on the basis of their geographical origin.

## 4. Conclusions

In this study, a rapid, easy-to-perform and low-cost method based on DART-HRMS analysis combined to multivariate statistical analysis was successfully developed and applied to classify monofloral honeys for their geographical origins, such as Italy and Portugal for chestnut samples and Italy and China for acacia samples. Specifically, three supervised approaches—i.e., PCA-LDA, PLS-DA and k-NN were evaluated. All tested models provided high and comparable recognition and prediction abilities in cross-validation and external validation, with mean values ranging from 89.2% and 98.4%. The performances of the proposed DART-HRMS method makes it an effective tool to assess the authenticity of honeys, for both industries of sector against unfair advantages of competitors and control bodies to fight food frauds. Future efforts will be directed to improve the current predictive models in order to discriminate honey samples from different production seasons and identify potential markers useful for developing a DART-HRMS target method aimed at honey authentication. Moreover, the use of the DART-HRMS approach, generating huge information in a single run, would be a useful tool for discriminating honey samples with similar organoleptic characteristics but different quality levels.

## Figures and Tables

**Table 1:** Model performances in terms of recognition, cross validation (CV) prediction abilities and external prediction to classify chestnut honeys based on their geographical origin.

	Model Performance (%)								
	Recognition Ability (Modelling)			Prediction Ability (CV ^c^ 10)			External Prediction		
	ITA ^a^	POR ^b^	Mean	ITA	POR	Mean	ITA	POR	Mean
**PCA/LDA ^d^** (7 Principal Components)	100.0 (30/30)	96.7 (29/30)	98.4	100.0 (30/30)	96.7 (29/30)	98.4	88.9 (8/9)	91.7 (44/48)	90.3
**PLS-DA ^e^** (10 Latent Variables)	100.0 (30/30)	96.7 (29/30)	98.4	100.0 (30/30)	93.3 (28/30)	96.7	88.9 (8/9)	89.6 (43/48)	89.2
**k-NN ^f^** (k value of 3)	96.7 (29/30)	100.0 (30/30)	98.4	96.7 (29/30)	100.0 (30/30)	98.4	88.9 (8/9)	93.8 (45/48)	91.4

^a^: Italy; ^b^: Portugal; ^c^: Cross Validation; ^d^: Principal Components—Linear Discriminant Analysis; ^e^: Partial Least Squares—Discriminant Analysis; ^f^: k-nearest neighbors.

**Table 2:** Model performances in terms of recognition, cross validation (CV) prediction abilities and external prediction for all models built to classify acacia honeys based on their geographical origin.

	Model Performance (%)								
	Recognition Ability (Modelling)			Prediction Ability (CV ^c^ 10)			External Prediction		
	ITA ^a^	CHI ^b^	Mean	ITA	CHI	Mean	ITA	CHI	Mean
**PCA/LDA ^d^** (9 Principal Components)	96.7 (29/30)	93.3 (28/30)	95.0	96.7 (29/30)	90.0 (27/30)	93.4	89.6 (43/48)	88.9 (8/9)	89.2
**PLS-DA ^e^** (12 Latent Variables)	100.0 (30/30)	93.3 (28/30)	96.7	96.7 (29/30)	93.3 (28/30)	95.0	93.8 (45/48)	77.8 (7/9)	85.8
**k-NN ^f^** (k value of 3)	100.0 (30/30)	90.0 (27/30)	95.0	93.3 (28/30)	90.0 (27/30)	91.7	91.7 (44/48)	88.9 (8/9)	90.3

^a^: Italy; ^b^: Portugal; ^c^: Cross Validation; ^d^: Principal Components—Linear Discriminant Analysis; ^e^: Partial Least Squares—Discriminant Analysis; ^f^: k-nearest neighbors.
